# Repair of Aberrant Right Subclavian Artery Causing Dysphagia Lusoria via Partial Median Sternotomy

**DOI:** 10.1177/21501351221137826

**Published:** 2022-11-15

**Authors:** Kevin R. An, Mimi X. Deng, Lindsay R. Freud, Osami Honjo

**Affiliations:** 1 Division of Cardiovascular Surgery, Labatt Family Heart Centre, Hospital for Sick Children, 7938 University of Toronto, Toronto, Ontario, Canada; 2 Division of Cardiology, Labatt Family Heart Centre, Hospital for Sick Children, 7938 University of Toronto, Toronto, Ontario, Canada

## Abstract

While unusual, aberrant right subclavian artery (ARSCA) can occasionally be a source of significant dysphagia in children. We present a case of a 13-year-old female who reported a three-year history of dysphagia to solid foods and was found to have ARSCA on a barium swallow study and computed tomography scan of the chest. We reimplanted the ARSCA into the right carotid artery in end-to-side fashion using a partial median sternotomy approach. At six months follow-up, her symptoms had completely resolved, and her postoperative echocardiogram showed an unobstructed reimplanted ARSCA. Meeting presentation: AATS 102nd Annual Meeting; May 14, 2022; Boston, MA.

## Introduction

Aberrant right subclavian artery (ARSCA) is a congenital anomaly with a prevalence of 1.2% to 2.2% in the general population.^1^ ARSCA usually does not cause symptoms as it does not form a complete vascular ring. However, in selected cases, the artery can cause posterior esophageal compression due to limited space between the esophagus and spine.

## Clinical Summary

**Figure 1.**
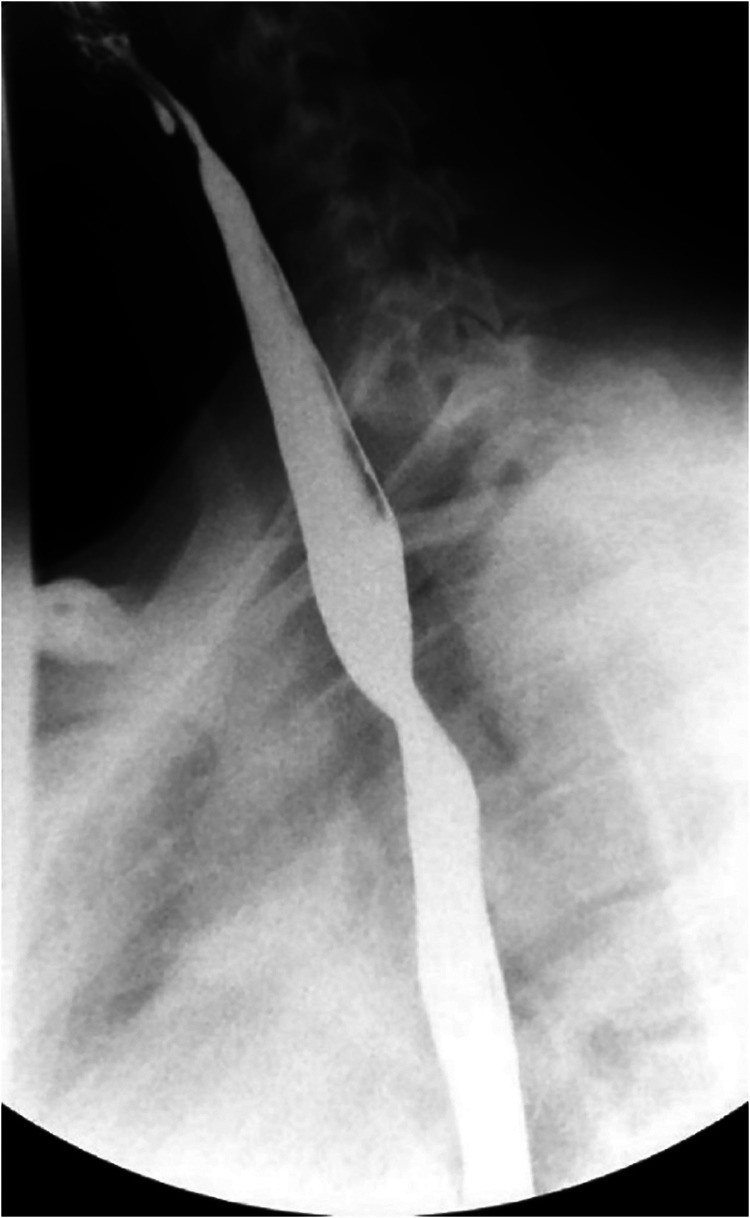
Barium swallow study demonstrating a mid-thoracic esophageal compression.

**Figure 2.**
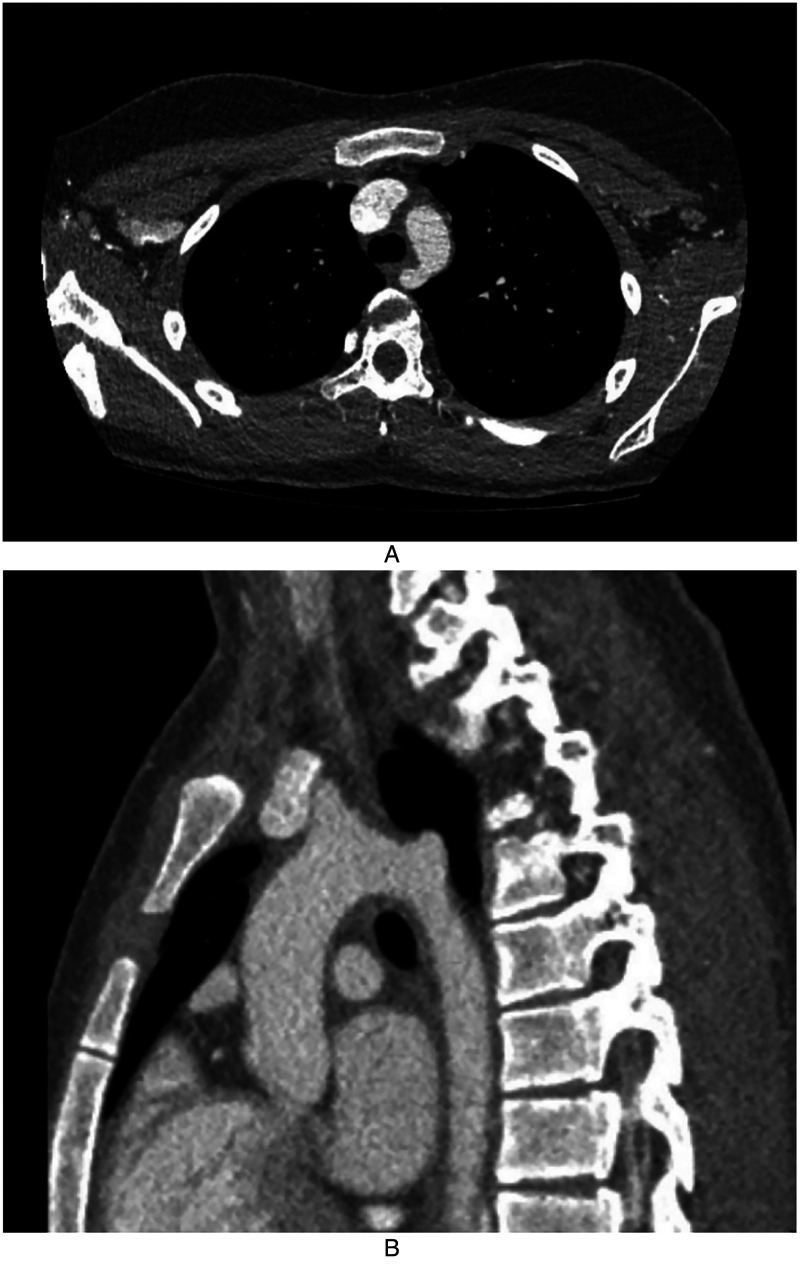
A and B, Computed tomography scan of the chest in axial (left) and sagittal (right) views demonstrating the right subclavian artery emerging from the left aortic arch coursing posteriorly behind the esophagus without a Kommerell diverticulum (left).

A 13-year-old female presented with a three-year history of dysphagia to solid foods. She occasionally regurgitated or vomited solid foods that she could not swallow. She had a history of velopharyngeal insufficiency with a posterior pharyngeal flap. A barium swallow study noted a mid-thoracic esophageal compression concerning for aberrant right subclavian artery (Figure 1). Computed tomography scan of the chest confirmed an ARSCA arising from the left aortic arch as the fourth branch coursing posteriorly behind the esophagus without a Kommerell diverticulum (Figure 2A and B). The proximal part of the ARSCA was more dilated than usual, which could have caused her symptoms. The challenges of this case involved the decision to pursue operative or medical management as well as the choice of surgical approach. Written informed consent for publication was obtained from the patient.

## Surgical Technique

**Figure 3.**
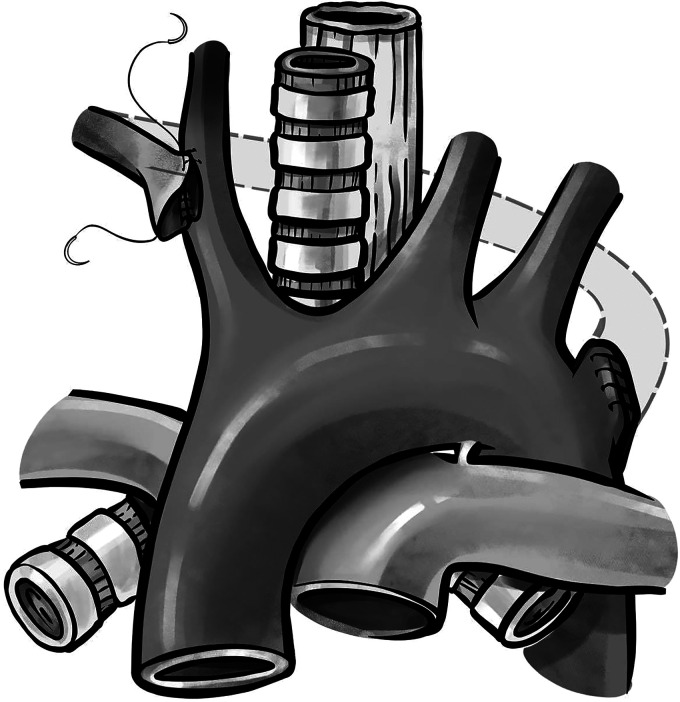
Operative diagram showing the reimplantation of the aberrant right subclavian artery into the right carotid artery.

A partial upper median sternotomy to the third intercostal space, without a J- or T-shaped sternal incision, was performed to reimplant the ARSCA to the right carotid artery. In children, a J- or T-shaped sternal incision is not necessary due to the flexibility of the sternum, and a central sternal split of the manubrium and upper sternal body is adequate for exposure. The dissection was continued along the posterior aspect of the aortic arch, until the ARSCA was identified and encircled at its base with a vessel loop. Identifying the artery's path right of the esophagus was more challenging due to its posterior position. By dissecting along the superior border of the innominate vein and along with the right lateral border of the trachea and esophagus, the distal extent of the right subclavian artery was identified and encircled. With proximal and distal control obtained, the ARSCA was clamped and divided at its proximal extent. The aortic arch was oversewn, and the artery was brought anterior to the esophagus and trachea by dissecting the soft tissue surrounding it. After the administration of heparin, a partial clamp was placed on the right carotid artery. The right subclavian artery was beveled and anastomosed to the right carotid artery in end-to-side fashion (Figure 3). After de-airing maneuvers were performed, a strong ARSCA pulse was felt. The chest was closed in routine fashion with one chest tube in-situ. The patient tolerated the surgery well and was discharged on postoperative day two. At six months follow-up, her symptoms of dysphagia had resolved and her postoperative echocardiogram showed an unobstructed reimplanted ARSCA.

## Comment

First reported by Bayford in 1794 and repaired by Gross in 1946, ARSCA is a common congenital anomaly that usually does not cause symptoms.^2^ When symptoms of dysphagia occur, it is termed “dysphagia lusoria.” Symptoms in children are usually due to the vessel appearing taut across the posterior esophagus, causing compressive symptoms, while symptoms in adults are more often due to age-related atherosclerotic changes or aneurysmal dilation, termed Kommerell diverticulum. In symptomatic children without a Kommerell diverticulum dietary modification with soft foods or smaller bites may be attempted if symptoms are mild, although this is controversial.^3^ In our case, upon discussion at multidisciplinary case rounds, given the severity of symptoms and lack of another cause, the decision was to proceed with the operation.

There are a number of different surgical approaches to ARSCA. Previous reports have described supraclavicular, right or left thoracotomy, and median sternotomy approaches.^2,4–6^ We chose a partial median sternotomy approach due to the belief it would provide the optimal exposure for accessing both the base of the right subclavian and the right carotid artery for end-to-side anastomosis. It is important to ensure that the vascular stump is not left too long and there is no persisting ligamentum arteriosum or dysphagia symptoms that may not resolve.^6^ Long-term outcomes of repair in children are good with one study reporting no long-term need for reoperation in 56 patients undergoing repair.^7^ Our case demonstrates isolated ARSCA as the cause of significant dysphagia in a child with complete resolution of her symptoms by relocation and reimplantation of her ARSCA.
